# Classifying Measures of Biological Variation

**DOI:** 10.1371/journal.pone.0115312

**Published:** 2015-03-25

**Authors:** Hans-Rolf Gregorius, Elizabeth M. Gillet

**Affiliations:** 1 Büsgen-Institut – Forstgenetik und Forstpflanzenzüchtung, Universität Göttingen, Göttingen, Germany; 2 Institut für ökologische und Populationsgenetik, Göttingen, Germany; Estacion Experimental de Zonas Áridas (CSIC), SPAIN

## Abstract

Biological variation is commonly measured at two basic levels: variation within individual communities, and the distribution of variation over communities or within a metacommunity. We develop a classification for the measurement of biological variation on both levels: Within communities into the categories of dispersion and diversity, and within metacommunities into the categories of compositional differentiation and partitioning of variation. There are essentially two approaches to characterizing the distribution of trait variation over communities in that individuals with the same trait state or type tend to occur in the same community (describes differentiation tendencies), and individuals with different types tend to occur in different communities (describes apportionment tendencies). Both approaches can be viewed from the dual perspectives of trait variation distributed over communities (CT perspective) and community membership distributed over trait states (TC perspective). This classification covers most of the relevant descriptors (qualified measures) of biological variation, as is demonstrated with the help of major families of descriptors. Moreover, the classification is shown to open ways to develop new descriptors that meet current needs. Yet the classification also reveals the misclassification of some prominent and widely applied descriptors: Dispersion is often misclassified as diversity, particularly in cases where dispersion descriptor allow for the computation of effective numbers; the descriptor *G_ST_* of population genetics is commonly misclassified as compositional differentiation and confused with partitioning-oriented differentiation, whereas it actually measures partitioning-oriented apportionment; descriptors of *β*-diversity are ambiguous about the differentiation effects they are supposed to represent and therefore require conceptual reconsideration.

## Introduction

Over the past decades, a confusingly large number of measures have been proposed for the quantification of biological variation (for a selection merely of “diversity” indices see e.g. [1]), yet relatively few of them are commonly used. Even ecologists and population geneticists who are aware of the richness of this repertoire may be tempted to apply the same measures that everyone else does, arguing that this ensures comparability between investigations. While this is certainly true, the restriction to a few measures leaves other measures and their important underlying concepts unused. This situation is aggravated by the fact that some of the commonly used measures suffer from widespread misinterpretation. Moreover, when conceptual or terminological aspects of measures of biological variation are addressed, these are restricted to the notion of diversity as the only category (for more recent contributions see e.g. [2] or [3] or refer to specifically population genetic measures as in [4]).

In this paper, an attempt is made to extend coverage of measures of biological variation by showing that they can be conceptually distinguished into four primary categories of variation: dispersion, diversity, compositional differentiation between communities, and partitioning of variation. The specifically biological reasoning for this classification is provided in the next section. Besides biological reasoning, the focus is set on developing and specifying these categories in ways that allow proper assignment of known measures, or others to be yet developed, to the categories. To prevent possible misunderstanding, it should be emphasized that the term classification is applied here to the conceptualization of categories of measures rather than to the advancement of methods of assigning measures to given categories or classes. The conceptual emphasis also means that statistical problems of estimating or testing of measures from samples will not be pursued in this paper.

The present approach explicitly builds on the elementary ingredients of variation: frequencies (or more generally, quantitative representations of the presence) of types, and differences between the types. On a high level of generality, types are defined by the states of any trait of the members of a community. (For more explanation of trait, including examples, see the glossary in Appendix A.) A community, in turn, is conceived of as any collection or ensemble of organisms that are connected and delineated by specified ecological or reproductive factors (including functional species communities, populations as reproductive communities, etc.). It is shown that an appropriate definition of difference between types allows consistent extension of descriptors of variation of qualitative traits to descriptors of variation of quantitative traits and, by this, reveals the connections and transitions between the categories of variation.

Following a brief reminder of what characterizes biological variation as compared to other kinds of variation and their sources, the concept of difference is explained. On this basis, the four essential categories of biological variation are derived. A discussion of several important applications of this classification follows, from which suggestions for new types of measures are shown to arise. These applications concern the duality of differentiation among communities and among trait states, the importance of distinguishing dispersion and diversity aspects of dispersion effective numbers, the differentiation and apportionment approaches to partitioning trait variation in metacommunities, the (mis)classification of *G*_*ST*_ and its relatives, and the classification of phylogenetic diversity. Basic definitions are given in the glossary in Appendix A.

## Results and Discussion

### Characteristics of biological variation

What characterizes biological variation as opposed to other forms of (non-biological) variation and their sources? Biological variation can change, if the environmental conditions in which an organism and its community is embedded change. This dependence of the trait state (or phenotype) of specified traits of organisms on their genotypes and environments is basically characterized by norms of reaction. In systems theory, norms of reaction are defined as mappings that assign environmental conditions (independent or input variable) to trait states (dependent or output variable), where the constructive specification of the assignment (system state) is realized by the genotype (or idiotype) and its epigenetic (regulatory) state. Apparently, in most cases neither the environmental conditions nor the portion of the genome involved in the expression of an organism’s trait are amenable to exhaustive observation. This does not, however, detract from the significance of the idealizing concept of the reaction norm, which was initially proposed in 1909 by R. Woltereck [5] (also see [6] for historical context). It follows that variation in traits of organisms can be attributed to two effects, the genetic effect, which is intrinsically qualitative or discrete, and the environmental effect, which may be quantitative and potentially continuous.

Opportunities to separate these two effects increase with decreasing range of the norms of reaction and thus with increasing heritability of the trait variation. (This is the realm of quantitative genetics as specifically addressed in the book of Namkoong et al. [7].) While sufficient separation of genetic from environmental effects promotes evolutionary adaptation of communities, broad-ranged norms of reaction are a sign of physiological adaptation (see e.g. the books of Levins [8] or Brandon [9]). The extension of this basic idea to more complex and less rigorously contained (isolated) units of heredity, such as species or (more generally) monophyletic groups, is straightforward. The description and analysis of biological variation is therefore largely concerned with traits that are either under chiefly genetic control, and are thus of predominantly qualitative (discretely varying) nature, or that vary more or less continuously with a tendency to cluster. Herewith, clustering patterns are governed by both the genetic and the environmental variation realized in the community under study as well as by the genotype-environment associations (joint frequency distribution of genotypes and environments) and by the genotype-environment interactions (differential effects of genotype and environment on phenotype expression).

Particularly when clustering or isolation tendencies within communities become more pronounced, the case of community subdivision becomes relevant. Subdivisions, which can result from adaptational specialization or *differentiation* but also from random effects, can be considered as communities in their own right. Their totality then can be viewed as a metacommunity (i.e., totality of individuals from a set of communities). Indeed, the formation of metacommunities is a well-known means of adaptational response via the evolution of local adaptations, between which relevant functional (including reproductive) relations are sustained among the constituent communities (see e.g. [8]). In this case, studies of the distribution of biological variation must consider two sources of variation: within and between communities.

Considering this perception of biological variation, its measurement covers two basic levels, variation within individual communities, and the distribution of variation over communities or within a metacommunity. The former level is concerned with aspects relating to spread, or *dispersion*, and aspects relating to numbers of types or distinct clusters, commonly addressed in terms of *diversity*. The second level is characterized by differences in trait distribution between communities, as summarized under the term *compositional differentiation*, and by the partition of the total (metacommunity) variation into variation within and between communities, commonly referred to as *partitioning of variation*.

Calling these four types of measures *categories*, it will be shown in the following that most families of descriptors of biological variation, including newly suggested ones, can be classified into or derived from one of the four. In this context, the term *descriptor* is understood to emphasize the conceptual underpinnings from which the respective measure draws its validity, and by this enables comparison between a variety of situations or models. The validity of a descriptor is thus to be judged by its compliance with its concept. In the present paper, this judgement is argued to be most reliably based on the suggested classification and, by this, facilitates identification of descriptors that are misclassified or misnamed.

### Difference

The perception of variation requires the ability to recognize differences between entities (or objects) of observation. At the very beginning, any attempt to classify measures of variation should therefore be based on a comprehensive concept of difference. Clearly, no object differs from itself, and thus every object is indistinguishable from itself. As soon as two different objects are considered, their distinguishability refers to a specified trait and, for example, to the resolving capacity of the observational equipment used to identify the trait and to distinguish its states. Of relevance here is not only the complexity of the trait, including its composition of multiple subtraits, but also the level of resolution specified by the problem to be studied. Particularly the latter aspect may lead to situations where a trait (mostly multidimensional) is to be studied under different resolution criteria with the aim of detecting hierarchical organizations of biological variation into species, races, and ecotypes, for example. In essence, this implies that objects considered indistinguishable at one level may be distinguishable at a lower level.

Once any means of qualitative or quantitative distinction is defined, it must be clarified whether these means allow for *consistent distinction* of the objects under consideration. By “consistent”, it is understood that the indistinguishability of any two objects should not be invalidated by comparisons with any third object. Given any non-negative function *d* defined for all pairs of objects under consideration, including pairs consisting of the same object twice (in a matrix setting this would be the diagonal), two objects *x* and *y* are considered indistinguishable on the basis of this function if *d*(*x*, *y*) = 0, while they are considered distinguishable if *d*(*x*, *y*) > 0. Consistency of such a function, in order to qualify it as a measure of difference, then requires that indistinguishability of one object from another implies equal differences of both from any third object (introduced as the “equivalence condition” by Bock [10]). The elementary requirements on a (consistent) measure *d* of difference are therefore (letting *x*, *y*, *z* denote objects from a specified set):

(a) *d*(*x*, *y*) ≥ 0
(b) *d*(*x*, *x*) = 0
(c) *d*(*x*, *y*) = 0 implies *d*(*x*, *z*) = *d*(*y*, *z*) for all *z*

**Fig 1:**
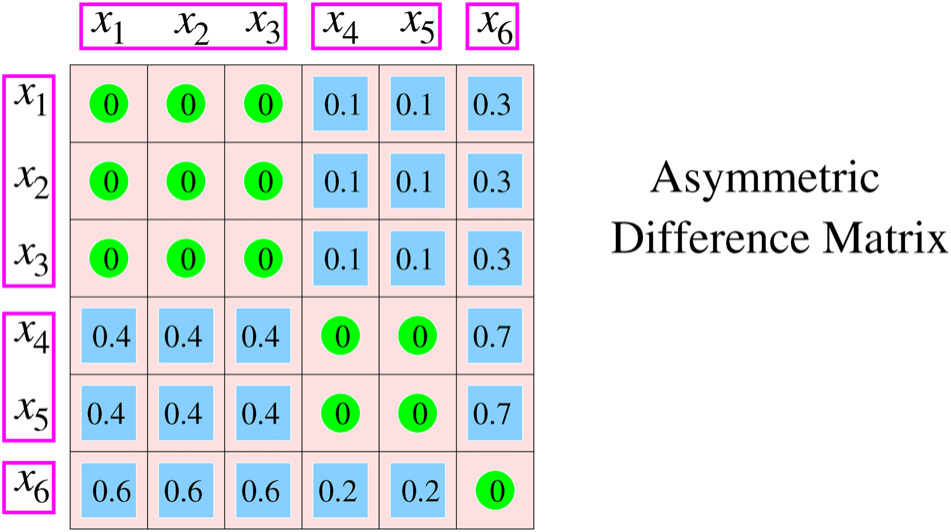
Difference. The asymmetric measure *d* of difference between objects *x*_*i*_ illustrated in matrix form is consistent, since it fulfills requirements (a) *d*(*x*, *y*) ≥ 0, (b) *d*(*x*, *x*) = 0, and (c) *d*(*x*, *y*) = 0 implies *d*(*x*, *z*) = *d*(*y*, *z*) for all *z*. The resulting equivalence classes are {*x*_1_, *x*_2_, *x*_3_}, {*x*_4_, *x*_5_}, and {*x*_6_}. The measure cannot distinguish between objects from the same class, but it can distinguish between objects from different classes. These three classes represent the primary partition of objects into the three types that are distinguishable by this measure.

Requirement (c) also implies that *d*(*y*, *x*) = 0 follows from *d*(*x*, *y*) = 0. Otherwise *d*(*x*, *y*) is not required to be symmetric, i.e., *d*(*x*, *y*) need not equal *d*(*y*, *x*) if both are positive. An illustration of the structure imposed especially by condition (c) on the matrix of differences is given in Fig. 1. There are many measures referred to as difference measures that are asymmetric. Examples are measures based on set differences where the difference of set A from set B is determined by the number of elements in A that are not in B. Several indices used in distinguishing DNA sequences are of this kind (for a survey see [11]). Yet, most of these do not meet the equivalence condition and are therefore not measures of difference in the above sense.

In the first place, measures of difference provide information on the way in which a collection of objects is subdivided into separated groups of indistinguishable objects. To see this, consider the relation ∼ between members of the collection that is defined by *x* ∼ *y* if *d*(*x*, *y*) = 0. This relation is reflexive (*x* ∼ *x*), symmetric, and transitive. The latter follows from requirement (c), which implies that *d*(*x*, *y*) = 0 and *d*(*y*, *z*) = 0 entail *d*(*x*, *z*) = *d*(*y*, *z*) = 0. Hence, *x* ∼ *y* and *y* ∼ *z* imply *x* ∼ *z*, which confirms transitivity of the relation and classifies it as an equivalence relation. The equivalence classes associated with the relation thus correspond to the groups of indistinguishable objects, and they form a partition of the collection that will be referred to as the *primary partition generated by the difference measure *d**. The primary partition can also be conceived as a trait with states (or types) given by the equivalence classes.

This property of difference measures becomes particularly relevant, when differences are measurable between objects without reference to any particular trait. In this case, the primary partition establishes a hitherto not realized trait. Moreover, any measure of “difference” between the states of a specific trait that does not obey the equivalence condition cannot be used in the detection of further trait structure hidden in the initial trait.

Among the host of difference measures, there is one family that is of special importance in that it sets an upper limit to difference and identifies this with the notion of complete distinctness of objects. This family will be referred to as *measures of dissimilarity*. It includes as a special subfamily measures of difference that assume only two values (zero indicating sameness, any other fixed positive value indicating differentness), known under the term *discrete metrics*. Recalling the above definition of the primary partition, discrete metrics can be used to characterize qualitative traits in the sense that two objects are either indistinguishable or completely distinct in their characteristics.

The transition from qualitative to quantitative traits becomes particularly evident when considering genetic traits, the states of which may be determined by the alleles at several gene loci (multilocus traits). Even though each component (locus) of such a trait is intrinsically qualitative, large numbers of components can result in almost gradual differences between the states of the (multilocus) genetic trait. While genetic types are recognized as being completely distinct if they share no alleles at the studied loci, the possible degrees of dissimilarity become ever more numerous as the number of loci increases. In a sense, the trait becomes more and more quantitative.

### Category: Dispersion

The more the members of a collection differ to larger extents from each other, the more dispersed or spread-out the variation pattern is judged to be. This roughly describes the dispersion aspect of variation. Therefore, dispersion measures are generally specified as

▹ non-negative functions of the differences between types and their frequencies, which
▹ equal zero only if all differences are zero, which
▹ do not decrease as differences increase, and which
▹ do not exceed the maximum difference realized.

Typical examples of dispersion measures are maximum differences and various kinds of average differences among collection members. The classics are thus max_*x*, *y*_ *d*(*x*, *y*) and ∑_*x*, *y*_ *p*_*x*_ ∙ *p*_*y*_ ∙ *d*(*x*, *y*), where *p*_*x*_ and *p*_*y*_ are the frequencies of the indexed objects or types. Particularly average differences are frequently considered to measure diversity, even though that term is usually used in the context of observations on discrete variables with a finite number of states (see e.g. the seminal paper of Rao [12]).

**Fig 2:**
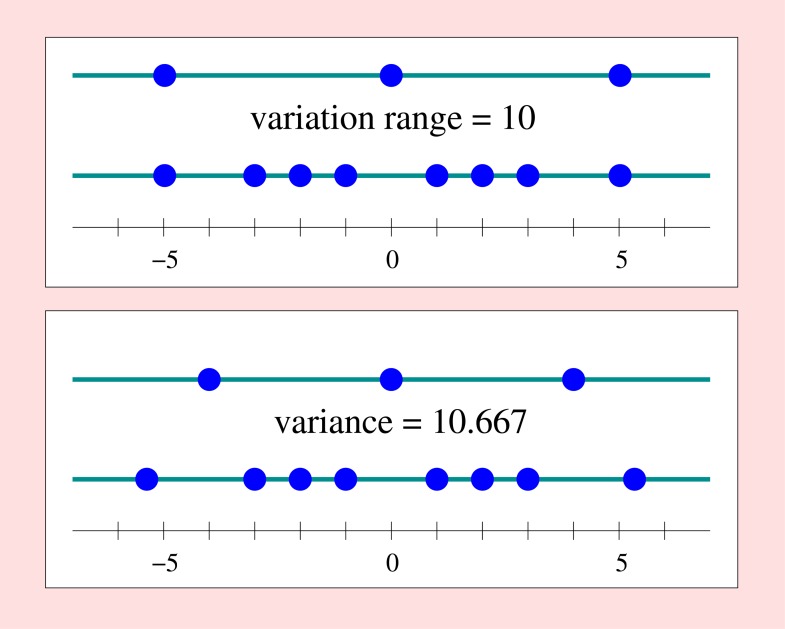
Dispersion. Two examples demonstrating that dispersion does not reflect the number of types. Upper frame: Defining dispersion as the variation range max_*x*, *y*_ *d*(*x*, *y*) for *d*(*x*, *y*) = ∣*x*−*y*∣, the smaller set {−5,0,5} has the same dispersion of 10 as the larger set {-5,-3,-2,-1,1,2,3,5}. Lower frame: Defining dispersion as the variance ∑_*x*, *y*_ *p*_*x*_ ∙ *p*_*y*_ ∙ *d*(*x*, *y*) with $d(x,y)=\frac{1}{2}{\left(x-y\right)}^{2}$, the smaller set {−4,0,4} has approximately the same dispersion of ca. 10.667 as the larger set {-5.354126, -3, -2, -1, 1, 2, 3, 5.354126}.

The number of types (or equivalence classes as given by the primary partition) that support an observed dispersion does not, however, explicitly show up in its measurement. This is illustrated in Fig. 2, where for each of two common measures of dispersion, the same value is realized in the presence of very different numbers of types (three and eight). The first measure, the variation range, is given by max_*x*, *y*_ *d*(*x*, *y*), where *d*(*x*, *y*) = ∣*x* − *y*∣. The second measure, the variance, has the form ∑_*x*, *y*_ *p*_*x*_ ∙ *p*_*y*_ ∙ *d*(*x*, *y*) with $d(x,y)=\frac{1}{2}{\left(x-y\right)}^{2}$.

In fact, consideration of numbers of types implies a change in the aspect of variation, and this requires specification of the conditions under which the transition between the two aspects is feasible. Since the counting of types assumes that they are unambiguously distinguishable, the pertinent difference measure is the discrete metric, as explained above. This, in turn, requires a decision on which difference value may ideally characterize distinctness of individuals. There are at least two ways to achieve this goal. An obvious way is to choose the maximum difference realized in the collection to characterize complete distinctness. If the difference measure is a measure of dissimilarity, the situation of complete distinctness is implied by the measure itself, and this is independent of the respectively realized differences.

Given an appropriate discrete metric, the last obstacle on the way to an unambiguous counting of types is presented by variable frequencies among the types. Removing this obstacle by assuming equal frequencies, dispersions can be computed for the accordingly defined ideal situation, in which the dispersion depends on the number of types only. Equating the ideal to the observed dispersion value and solving the equation for the number of types yields the desired relation between measures of dispersion and numbers of types supporting the dispersion.

**Fig 3:**
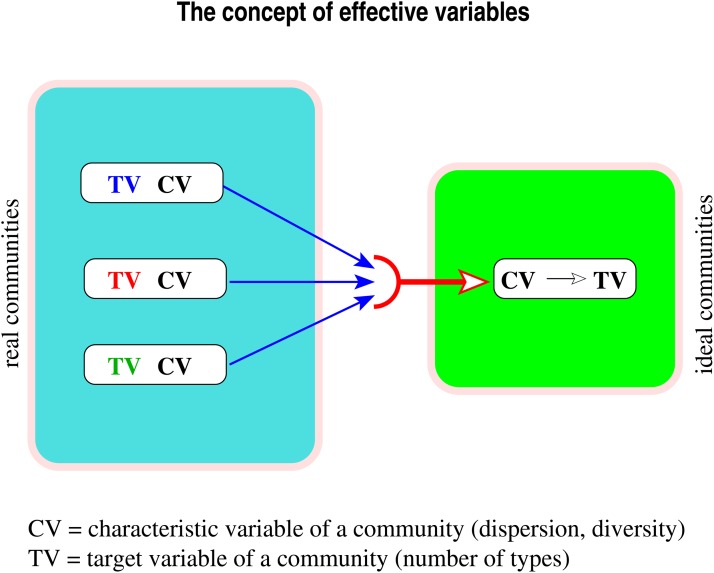
Effective variable. Diagram of the ingredients of the concept of effective variable and their relationships. Left: Three (real) communities that are identical in their characteristic variable (CV) but differ in their target variable (TV). Right: The ideal community that equals the real communities in its characteristic variable; the uniquely specified target variable of the ideal community summarizes the values of the target variable of the real communities into a single *effective* value.

In essence, the above procedure repeats the steps to be taken when computing “effective numbers” according to the underlying general concept of Gregorius [13]. Since “effective number” is a notion that is more familiar in ecology and population genetics than in other fields such as economics, the following explanations will use the term community in place of collection, even though in many respects the more comprehensive term collection would also be appropriate. When applied to measures of dispersion as the *characteristic variable* of communities and the number of types as the *target variable* of communities, the concept requires definition of *ideal communities* (a) that realize all dispersion values occurring among the (non-ideal) communities under consideration, and (b) in which the number of types is a strictly increasing function of the dispersion value. The *effective number* (i.e. the effective value of the target variable) then results from equating an observed dispersion with the dispersion of an ideal community (see below) and solving for the number of types in the latter. Since this number refers to dispersion characteristics, it is termed *dispersion effective number of types* in accordance with common practice. For an illustration of the concept see Fig. 3.

What is an ideal community for dispersion? Requirement (b) indeed implies that in ideal communities, different types are not allowed to vary in their difference measures nor in their frequencies. This follows from the fact that the same dispersion can be realized for various combinations of differences and frequencies of types; such possibilities must be excluded from an ideal community in order to establish a unique assignment of dispersion values to number of types. Hence, all types in the ideal community have equal frequencies, and the corresponding dispersion measure is a discrete metric. Therefore, since by requirement (a) dispersions cannot exceed the upper value of the discrete metric, the maximum value of all differences equals that within the ideal communities. As a consequence, *a dispersion effective number of types exists only for difference measures that are dissimilarity measures*. Yet not all dispersion measures based on dissimilarities allow for the computation of effective numbers. Examples are listed in Table 1.

**Table 1:** Dispersion measures with no effective number.

▹	max_*x*, *y*_ *d*(*x*, *y*)
▹	$\sum_{x}p_{x}∙(\sum_{y:y\neq x}p_{y}∙d(x,y)/(1-p_{x}))$
▹	$\sum_{x\neq y}p_{x}∙p_{y}∙d(x,y)/(1-\sum_{z}p_{z}^{2})$

An effective number does not exist for the following measures of dispersion, since they do not fulfill requirement (a) of the concept of effective number

Replacement of dissimilarities by their complements, i.e., *similarities*, converts measures of dispersion into *measures of concentration*. The procedure leading to the definition of the dispersion effective number applies identically to similarities and thus to the definition of the *concentration effective number*. An example of a family of dispersion measures and its corresponding family of concentration measures together with their respective effective numbers is given in Table 2.

**Table 2:** Dispersion measures with effective numbers.

Dispersion measure ${}^{a}D≔{\left(\sum_{x}p_{x}∙{\left(\sum_{y}d(x,y)∙p_{y}\right)}^{a-1}\right)}^{\frac{1}{a-1}}\;(0\leq a\neq 1)$
^*a*^ *D*_*ideal*_ = 1 − *n* ^−1^ ⇒ dispersion effective number = (1 − ^*a*^ *D*)^−1^
For *a* = 2, where ^2^ *D* = ∑_*x*, *y*_ *p*_*x*_ ∙ *p*_*y*_ ∙ *d*(*x*, *y*), see [41]
Concentration measure ${}^{a}C≔{\left(\sum_{x}p_{x}∙{\left(\sum_{y}s(x,y)∙p_{y}\right)}^{a-1}\right)}^{\frac{1}{a-1}}\;(0\leq a\neq 1)$
^*a*^ *C*_*ideal*_ = *n* ^−1^ ⇒ concentration effective number = ^*a*^ *C* ^−1^ [42]

Effective numbers for a family of dispersion measures (^*a*^ *D*) and a family of concentration measures (^*a*^ *C*) based on a dissimilarity measure 0 ≤ *d*(*x*, *y*) ≤ 1 and a similarity measure *s*(*x*, *y*) = 1 − *d*(*x*, *y*), respectively; *n* ≔ number of types.

Effective numbers can thus be obtained for some but not all measures of dispersion. Effective numbers (or number equivalents) are likewise essential in the conceptualization of diversity indices, as will be demonstrated in the next section.

### Category: Diversity

In view of the overwhelming amount of work devoted to the concept of diversity and its variants, it is mandatory to first recall a basic feature of diversity measurement that seems to be widely agreed upon and then to see how this can be consistently extended to cover common notions. In the first place, and contrasting with the concept of dispersion, diversity aims at quantifying the heterogeneity of a community with respect to a qualitative trait, the states of which are equally distinct by definition. Using already introduced terminology, this implies that variation be characterized by a discrete metric, as variable differences between types are not at issue. The probably most obvious way to assess the heterogeneity of a community then consists in counting how many types of individual occur in the community. At this stage, frequencies of types are of no concern. As separate from dispersion, type counts therefore characterize the second of the two aspects of variation referred to in the section on biological variation. Type counts can be considered to constitute the *intrinsic concept of diversity*. The types of primary interest in community ecology and population genetics are species and alleles, respectively, and the corresponding type counts are commonly termed species richness and allelic richness.

Mere type counts may lose biological and statistical import when the representation of types in a community varies distinctly. Representation criteria may be determined, for example, by the number of community members showing a type, the area occupied by carriers of a type, the total biomass of the carriers of a type, the average similarity of a type to all community members, the average difference of a type from other types together with its frequency, or the frequency of individuals that do not differ by more than a given degree from some specific type. All of these criteria and their implied weighting of types affect the intrinsic concept of diversity only via their relative import and thus, after normalization, yield sums of weights equal to one. Ideally, however, the intrinsic concept of diversity would be realized only when all types are represented equally. Consequently, when the number of equally represented types is increased, any measure of diversity should also increase, and it should decrease when types become less equally represented.

These demands on measures of diversity consistently extend the intrinsic concept, in that they stress the closeness to an even representation of types as a principle by which diversity can be increased. This can be clad into what can be called an *evenness criterion* to be posed on legitimate measures of diversity, and which has been given different formulations and different names (such as “principle of transfers”, see e.g. [14] or [15]). Concisely, the condition requires that *diversity never decreases whenever the difference in representation between two types decreases while the sum of their representations remains the same*. To avoid confusion with other notions such as “component of diversity” to be discussed later on, the addition “in the strict sense” will be used where appropriate to distinguish measures of diversity that meet the evenness criterion.

Given this extension of the term “diversity”, a direct connection to its intrinsic concept can be established with the help of the previously introduced notion of effective numbers. In the present context, the characteristic variable is the respective measure of diversity, the target variable is the number of types, and in the ideal communities all types are equally represented. Requirements (a) and (b) of the concept of effective number are obviously fulfilled, so that a *diversity effective number* of types is unambiguously defined. Because of the monotonicity in requirement (b), this effective number fulfills the evenness criterion and is thus again a diversity measure. Some common and a few less common examples of diversity measures and their effective numbers are listed in Table 3 (for a more comprehensive account see e.g. [14, 16]; these authors also refer to the effective number as the “numbers equivalent”).

**Table 3:** Diversity effective numbers.

Diversity measure		Diversity effective number
∑_*i*_ *p*_*i*_ ∙ *r*(*p*_*i*_)		1/*r* ^−1^(∑_*i*_ *p*_*i*_ ∙ *r*(*p*_*i*_))
*p* ∙ *r*(*p*) is concave ^(1)^		if *r* is strictly decreasing
**— Special cases —**		
	*r*(*p*)	
$1-\sum_{i}p_{i}^{a},a>1$	1−*p* ^*a*−1^	${\left(\sum_{i}p_{i}^{a}\right)}^{\frac{1}{1-a}}$ ^(2)^
$\sum_{i}p_{i}^{a},0\leq a<1$	*p* ^*a*−1^	${\left(\sum_{i}p_{i}^{a}\right)}^{\frac{1}{1-a}}$ ^(2)^
−∑_*i*_ *p*_*i*_ ∙ log_*a*_ *p*_*i*_, 0 < *a* ≠ 1	−log_*a*_ *p*	$\prod_{i}p_{i}^{-pt}$ ^(2)^
∑_*i*_ *p*_*i*_ ∙ (1 − *p*_*i*_)^*a*^, 0 < *a* ≤ 1	(1 − *p*)^*a*^	${\left(1-{\left[\sum_{i}p_{i}∙{\left(1-p_{i}\right)}^{a}\right]}^{\frac{1}{a}}\right)}^{-1}$
$\sum_{i}p_{i}∙\cos(p_{i}∙\frac{\pi }{2})$	$\cos(p∙\frac{\pi }{2})$	$\frac{\pi }{2}/\arccos(\sum_{i}p_{i}∙\cos(p_{i}∙\frac{\pi }{2}))$

^(1)^ see Appendix B for a proof of the evenness criterion

^(2)^ also called Hill numbers [43] or Rényi diversity [44]

Examples of diversity measures and their effective numbers; *p*_*i*_: = relative representation of the *i*-th type (only *p*_*i*_ > 0 considered; *r*(*p*) is a non-negative function of *p* (0 < *p* ≤ 1)

### Diversity for variable differences

The possibility of uniquely characterizing equally distinct types by discrete metrics hints at the possibility that some measures of dispersion, when applied to discrete metrics, may acquire the characteristics of a measure of diversity. Yet, attempts to further extend the intrinsic concept of diversity to include variable differences, as is the normal case for dispersion measures, encounter the problem that the condition of distinctness of types may not be met and that thus the evenness criterion (see glossary) no longer applies. The question is thus whether and how the condition of distinctness of types and the evenness criterion can be restored under variable differences.

The specifically biological motivation behind this question becomes clear when recalling the characterization of biological variation in terms of norms of reaction. These norms basically involve genetic types and thus qualitative sources of trait variation. The trait variation may be describable on a metric scale, but in each environment the variants are distinct to the degree to which the genetic effects penetrate (are expressed). Characteristics of a qualitative trait are thus retained. As the environment varies, the trait expressions of the genetic types may also vary, assigning to each genetic type a range of trait variants. In combination with genotype-environment associations and interactions, these ranges may overlap, blurring the distinction between genetic types. As a result, when plotting the frequency distribution of the trait variation over that variation, multimodality of the distribution (i.e., a number of separate peaks) will often show up, especially if different types dominate in different environments. Multimodality in trait distribution therefore mirrors the qualitative variation in the underlying genotypes and possibly the environmental conditions. Trait variation is thus no longer strictly distinct, but it would be discontinuous. Thus the diversity category remains relevant.

Statements on distinctness, or distinguishability, ideally entail threshold levels of difference (levels of resolution), above which objects are perceived to be different. An indispensable step towards extension of the intrinsic concept of diversity therefore asks for methods that yield a partition of a community into classes, such that two objects belonging to different classes have a difference that exceeds the threshold level, while objects within the same class do not. Such methods are well known from the field of cluster analysis. Careful selection of the clustering method may even give rise to transformations (the so-called cophenetic differences) of the actual differences between types that more closely reflect desirable aspects of “neighborhood” or distinctness than do the actual differences. In a dendrogram, for example, the cophenetic difference between two types is measured as the height of the smallest cluster containing the two types. Such transformed differences serve as the basis for further, more directed, diversity analyses.

**Fig 4:**
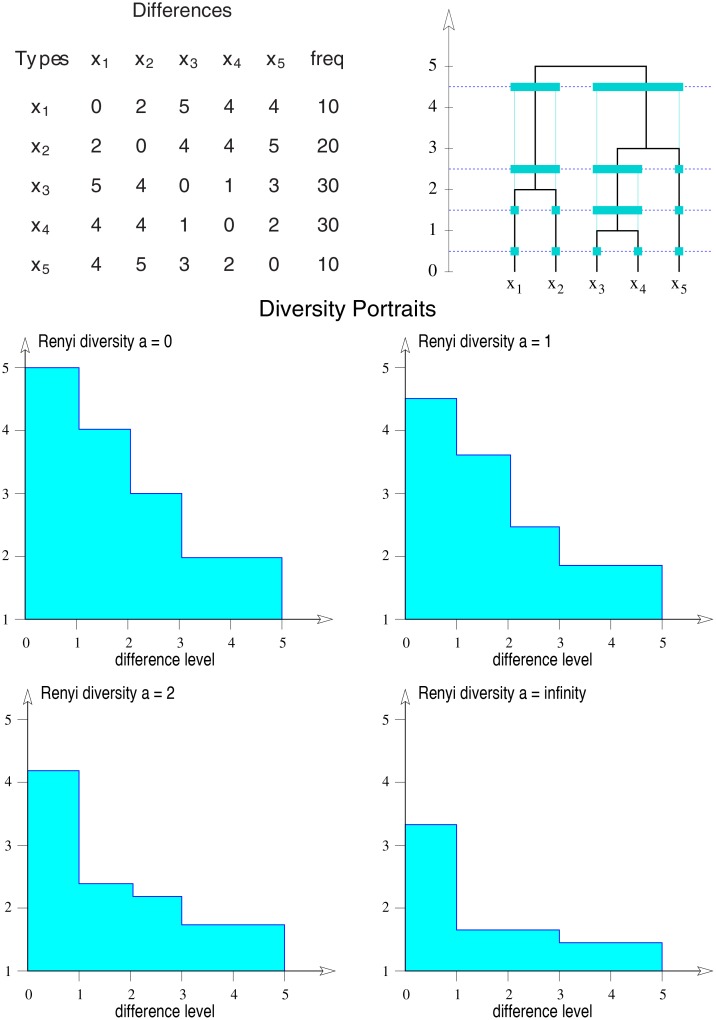
Diversity portraits. Top: Difference matrix of five types and the corresponding clustering of types for increasing difference level (y-axis). In addition to the primary partition, three hierarchically organized higher order partitions arise. Bottom: Four diversity portraits based on the above difference matrix showing Rényi diversity of different orders *a* as functions of difference level. In all cases, the diversity function shows a stepwise decrease as the difference level increases (see [17]).

Each partition obtained from an appropriate clustering method can then be considered as a trait with states given by the classes and state frequencies given by the sizes of the classes. In this way, the condition of distinctness of types and the evenness criterion are applicable, and measures of diversity as well as their effective numbers are defined for each level of resolution. Plotting the diversity of each partition against its corresponding level of resolution (clustering level) yields *diversity portraits* in the form of decreasing step functions (for an example see Fig. 4). The decreasing form of the portrait follows the form of the hierarchical ordering of the partitions and is implied by the evenness criterion. For more detailed explanations and interpretations of diversity portraits see [17].

Even though diversity effective numbers and dispersion effective numbers refer to the same ideal communities (discrete metric, equal type frequencies), they should not be confused. The two kinds of measure are distinguished by the requirements of the evenness criterion. As Pavoine et al. [18] showed in some detail, dispersion measures are maximized for equal type frequencies only for very special kinds of difference metrics (such as ultrametrics). Application of the evenness criterion to dispersion measures is thus not meaningful, unless the underlying differences imply a partition of the community into classes (as ultrametrics do). Even in these special cases of difference metrics, it should be kept in mind that the two effective numbers, though equal in value, are descriptors of different concepts and different categories of variation.

As was mentioned above, an alternative approach to the consideration of variable differences or similarities between types in a diversity context consists in regarding the difference of a type from other types as a contribution to its representation. An example is provided by multiplication of a type’s frequency by its average difference from the other types (as is implicit in the second example of Table 1). Normalization by the sum over types (as appears as a measure of dispersion in the example of Table 1) then yields the required relative representation of the types. In this case, the representation of a type increases with its frequency and its isolation from other types (the latter measured by the average difference). Any legitimate measure of diversity can then be applied to this type representation with an appropriate interpretation. For diversity effective numbers, such an interpretation could be in terms of an effective number of isolated types.

### Category: Compositional differentiation

Differences between whole communities are the subject of interest in studies of compositional differentiation in metacommunities. The objects to be distinguished are thus communities, and the relevant aspect of variation consists in the differences in trait distribution between communities. Amounts of variation within the individual communities as well as in the total metacommunity are not of primary relevance. Obviously, there are two tiers of difference involved in the creation of compositional differentiation:

▹ differences in type between individual members of the metacommunity,
▹ differences between communities, whereby this second tier depends on the first.

Moreover, a major and pervasive conceptual characteristic of compositional differentiation is to be found in the perception that the differentness among communities cannot exceed a state in which they share none of the attributes under consideration, irrespective of the quality of the attributes. This identifies measures of compositional differentiation as measures of dissimilarity in the above sense. Since complete dissimilarity among communities in turn presumes a perception of complete differentness between individual members of the metacommunity, one concludes that the concept of compositional differentiation involves measures of dissimilarity at both tiers of difference.

So far, complete distinctness of two communities for a given measure of the difference in type is equivalent to the complete distinctness of any member of either community from all members of the other. The other extreme, the identity of two communities, can be unambiguously depicted as follows: Consider the metacommunity that comprises only these two communities and build the primary partition of the individuals within this metacommunity for their types. Intersect the partition classes with the one and then the other community to obtain the frequency of each class (type) in the respective community. Identity between the two communities of the relative frequencies of each class indicates the absence of differentiation. For a qualitative trait, this simply states that the frequency distributions of the types are identical for both communities.

The extension of compositional differentiation to multiple communities then follows the lead provided earlier for measures of dispersion. The underlying objects are now communities, and their pairwise dissimilarities are based on the individual dissimilarities as explained in the previous paragraph. The primary partition generated by the individual dissimilarities is to be determined for the metacommunity consisting of all communities, and the distribution of the resulting classes in each community is obtained from intersection of the classes with the respective community. Some dispersion measures yield measures of compositional differentiation, such as the last two indices listed in Table 1, where *d*(*x*, *y*) is the dissimilarity between communities *x* and *y*, and *p*_*x*_ is the relative size of community *x*. In contrast, the dispersion measure ^2^ *D* listed in Table 2 cannot be turned into a measure of compositional differentiation, since the value it assumes for completely dissimilar communities varies with the sizes of the communities.

**Fig 5:**
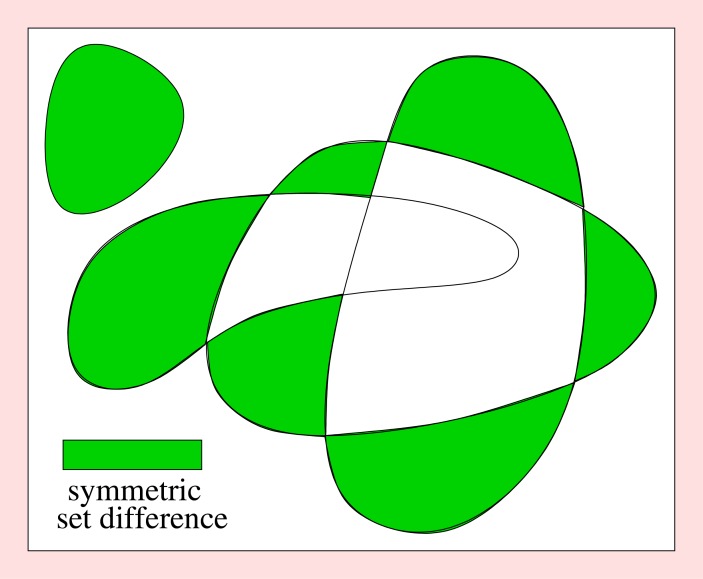
Compositional differentiation. Shaded areas represent the symmetric difference between four sets (closed curves), illustrating the extent of compositional differentiation via community characteristics of any one set that is not shared with its respective complement (modified from [40]).

Another type of measure of compositional differentiation summarizes the differences of each community from its respective complement in the metacommunity. Reminiscent of the symmetric set difference (see Fig. 5 for an illustration), it takes the form Δ_*SD*_ = ∑_*x*_ *P*(*C* = *x*) ∙ Δ(*C* = *x*, *C* ≠ *x*), where *P*(*C* = *x*) denotes the relative representation of community *x*, and Δ(*C* = *x*, *C* ≠ *x*) denotes the difference between community *x* and its remainder (*C* ≠ *x*) in the metacommunity. Note that Δ_*SD*_ is not a dispersion measure in the sense that it increases with the dissimilarities between any two communities. It increases only if communities become more dissimilar from their respective remainder in the metacommunity. In fact, Δ_*SD*_ can be consistently derived in a way that is not driven by ideas of variation but rather that considers individuals to be assigned to communities according to their types. Δ_*SD*_ is then seen to constitute a measure of association of community membership with trait state [19].

Any measure of compositional differentiation critically depends on the way in which the underlying type differences and their distributions are transformed into community differences. Among the many conceivable ways, one is of central relevance in that it specifies the minimum amount of change in type differences required in the trait distribution of one community to make it match the trait distribution in another community. Details of the conceptual justification and computational implementation of minimum differentiation are provided in [20]. In the special case of discrete metrics with values 0 and 1, the minimization principle results in the familiar measure $\Delta (p,q)\;=\frac{1}{2}\sum_{i}|p_{i}-q_{i}|$ of difference between two communities *p* and *q* with type frequencies *p*_*i*_ and *q*_*i*_, respectively. In ecology, 1−Δ is traditionally termed “percentage similarity” and is used in the quantification of *β*-diversity (see [21]). In population genetics, Δ is often called the “allele-sharing distance” [22, 23].

Variable differences can be subjected to cluster analysis to yield a partition of the metacommunity at any clustering level (level of resolution), as was introduced in connection with the diversity category. The classes of a partition can be considered as types (states of a trait) that are distinguishable at the respective level of resolution. Since distinguishability is the only criterion separating the thusly created types, they can be represented by a discrete metric. For a given partition, the intersection of its classes with the communities (as considered above for the primary partition) yields the distribution of the classes within each community. To this situation, any measure of compositional differentiation can be applied that rests on discrete metrics. In the same way that diversity portraits are obtained, one arrives at a *differentiation portrait* by plotting the compositional differentiation of each partition against its corresponding level of resolution. By definition, differentiation portraits are preferable over the computation of a single value of the minimum differentiation whenever threshold differences between individuals are suspected to trigger the operation of differentiating forces.

### Duality of compositional differentiation

**Fig 6:**
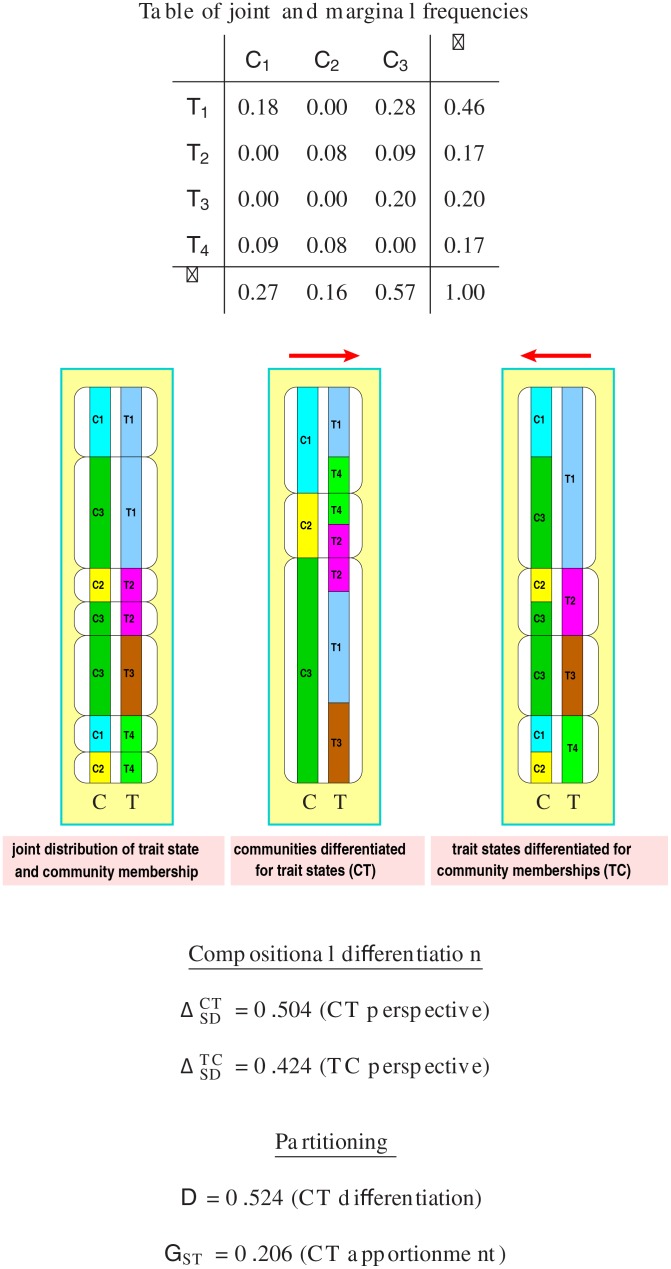
Perspectives of differentiation. Example of the dual perspectives of differentiation based on a qualitative trait *T* and community membership *C*. Top: Table of joint frequencies of the trait states and communities and the corresponding marginal frequencies. Columns refer to the CT perspective, rows to the TC perspective. Center: Illustrations of the joint frequency distribution and the distributions for the dual perspectives CT (sorted by communities, columns) and TC (sorted by types, rows). Arrows emphasize the direction of the perspective. Bottom: Values of the descriptors $\Delta_{SD}^{CT}$, $\Delta_{SD}^{TC}$ (compositional differentiation), *D* (partitioning-oriented differentiation), and *G*_*ST*_ (partitioning-oriented apportionment) for the frequencies in the table (for explicit specification of the descriptors, see text).

So far, compositional differentiation for a specified trait was viewed from the perspective of communities that differ for the distribution of the trait states (the CT perspective). The reverse perspective, where trait states are assessed for differences in the community membership of their carriers (the TC perspective) may seem odd, yet it is relevant in many biological studies, and there are even prominent cases where the two perspectives are confused. When writing the joint frequency distribution of trait and community membership in matrix form as in Fig. 6, CT refers to the columns and TC to the rows.

Plants with wind-dispersed seeds provide a typical example of the CT perspective, since the location of the parents has much more influence on the community in which a seed settles than its trait state or that of its parents, with the result that the communities are differentiated for the trait states of the seeds that reach it. A contrasting example of the TC perspective is given by mobile organisms, especially animals, that show differential disposition to stay in or migrate to special habitats, with the result that types are differentiated for the communities in which they dwell after migration [24].

From a mathematical point of view, these dual perspectives result by simply switching the two variables *T* and *C*, so that $\Delta_{SD}^{CT}$ would measure compositional differentiation of communities for a specified trait, while $\Delta_{SD}^{TC}$ would measure the respective differentiation of trait states for community membership (for an illustration of the two perspectives together with Δ_*SD*_ values, see Fig. 6).

The easiest way to identify cases in which the perspectives are confused is to describe their extremes. Thus, complete compositional differentiation of communities (CT perspective) implies that all carriers of the same trait state belong to the same community. Conversely, when trait states are completely differentiated for community membership (TC perspective), this implies that individuals showing different trait states cannot belong to the same community. From the CT perspective, communities are therefore monomorphic. This asymmetry in perspective vanishes in the absence of differentiation, since this case is characterized by stochastic independence between the variables *T* and *C*, so that differentiation is absent from both perspectives.

The TC perspective of differentiation in general probably becomes more familiar when it is described in terms of the *apportionment of variation*. Apportionment of trait variation to communities (which takes a CT perspective) describes the situation where individuals that *differ* in type tend to occur in *different* communities. At the extreme, where the trait variation is fully divided among communities, one again arrives at the situation of monomorphy within all communities. The CT perspective of apportionment therefore seems to be related to the TC perspective of differentiation [19]. Yet, this applies only at the extreme and does not allow the apportionment approach to be regarded as part of the concept of compositional differentiation. Instead, the apportionment approach belongs to a different category called “partitioning of variation” that will be treated in the next section. It will be shown there that confusion of the notions of differentiation and apportionment led to a far-reaching misnomer that triggered misconceptions and misinterpretations of a commonly used descriptor.

### Category: Partitioning of variation

Generally speaking, trait variation is considered to be partitioned, if it is divided among specified parts of a collective whole. The total variation can thus be perceived of as being composed of variation that exists within and between the parts. This perception differs fundamentally from that of compositional differentiation, for which neither the amount of total (metacommunity) variation nor the amount of variation within communities are primary determinants (see above). The partitioning notion governs much of biological theory on the distribution of organismic variability. It obtains its statistical support from the analysis of variance, which rests on the fact that the total variance of a random variable (trait) equals the sum of the expectation of the conditional variance (variation within) and the variance of the conditional expectation (variation between). The conditioning event is provided by a second random variable, such as community membership. Methods of extending this mathematical relationship to cover special measures of difference between more complex trait states were developed with reference to Euclidean spaces for example by Excoffier et al. [25] in a population genetic context and by Pavoine et al. [26] in an ecological context. The latter authors also related their approaches to ordination methods.

Variances and their generalizations to Euclidean spaces are special kinds of dispersion measure, and these differ conceptually from measures of diversity, as explained above. One can therefore suspect that the method of decomposing variances into components cannot be directly translated into measures of diversity, although this is essentially what Whittaker proposed in his seminal 1960 paper [21] by coining the terms *α*,- *β*- and *γ*-diversity. In fact, these terms and their transformation into measures remains a matter of lively discussion to this day. Among the three “diversities”, only *γ*-diversity seems to be generally accepted to unambiguously refer to the diversity of a collection. *α*-diversity, which is commonly conceived of as an “average” taken over the diversities realized within the collections that form the collective whole (communities of a metacommunity), is also considered by many researchers to be a reasonable proposition. However, besides the observation that an average of diversities is not a measure of diversity of any specific kind of collection, this passes over the fact that there are different kinds of averages, and that some of them do not accord well with the other two “diversities” (for one of the earliest reminders of this fact see [27]). In any case, *α*-diversity is inferred from the diversities measured within the communities, and by this it does not depend on differences in composition between communities.

Almost all researchers seem to agree with the idea that *β*-diversity should somehow reflect the compositional heterogeneity between communities. As recalled above, Whittaker [21] indeed proposed the “percentage similarity” as a measure of *β*-diversity. Its inverse, the “percentage dissimilarity”, is a measure of compositional differentiation between two communities. This confirms the idea of *β*-diversity, though surely not in terms of a measure of diversity. It also hints at an early problem concerning distinction between the two categories of biological variation: compositional differentiation and partitioning of variation. An impression of the underlying terminological problems can be obtained from the more recent reviews of Jurasinski et al. [28] and Jurasinski & Koch [3].

The desire to connect the three “diversities”, while preserving their conceptual motivations, leads to the central requirement that measures of *α*-diversity should not exceed measures of *γ*-diversity, and that both become equal only in the absence of differentiation among communities (for a concise statement of this requirement see e.g. [29]). This leaves *β*-diversity to explain and measure the difference between *α*- and *γ*-diversity as the result of differentiation, as is familiar from decomposition of the total variance (*γ*) into components of variance within (*α*) and between (*β*) collections. *β*-diversity is thus inferred from *γ*- and *α*-diversity, where *α*-diversity in turn is inferred from the diversities within communities. Since the assessment of differences between communities need not depend on diversities within communities, *β*-diversity may be considered to not depend on *α*-diversity (for more elaborate arguments on this view of independence of the two diversity components see [16]; in the next section, this topic together with the notion of “components of diversity” is resumed in a more comprehensive context).

Apparently, the first problem to be solved is to find a suitable kind of averaging over diversities. Especially for diversity effective numbers as provided by the family of Rényi-diversities (see Table 3), simple linear averaging fails. In this case a solution is provided for the Rényi-diversity of order *a* by the power mean of order (1−*a*) with weights given by the community sizes (see [30]; [31]). In connection with the multiplicative decomposition of *γ*-diversity into its *α* and *β* components, Jost [16] argues in favor of power means based on equal community sizes (which implies that *γ* is also to be determined for equal community sizes). There are in fact even more averages that satisfy the above central requirements on measures of *α*-diversity. It is straightforward to show that any average less than or equal to the power mean of order (1−*a*) also satisfies the requirements. Since power means increase with their orders, any power mean with order smaller than (1−*a*) is thus eligible for the measurement of *α*-diversity. One therefore concludes that the evenness criterion in combination with the central requirements on *α*-diversity does not suffice for the design of a canonical method of obtaining unique measures of *α*-diversity for each admissible measure of diversity. It is not even clear whether each admissible measure of diversity allows for a suitable measure of *α*-diversity.

However, once a suitable *α*-diversity has been identified for a diversity effective number, Jost [16] argued that (for Rényi-diversities) measuring *β*-diversity by the ratio of *γ*- to *α*-diversity allows for an interpretation of *β*-diversity as an “effective number of distinct communities”. Herewith it should, however, be recalled that neither *γ*- nor *α*-diversity nor any function of the two provide information on the state of complete differentiation between communities. Therefore care should be taken to not generally identify the idea of “distinctness” of communities with “completeness of differentiation”.

The differentiation aspect of *β*-diversity (which Jurasinski et al. [28] considered as a separate category of *β*-diversity) can be made more explicit under the above demands on *α*-diversity if one introduces the joint diversity *ϕ* of trait *T* and community membership *C*. *ϕ* is related to differentiation by the fact that *ϕ* ≥ *γ* holds, with equality only for complete differentiation [31]. Retaining the two extremal requirements on (compositional) differentiation (i.e., the absence of and complete differentiation), measures of *partitioning-oriented differentiation* ranging between 0 and 1 can be designed in various ways [31]. One such measure is of the general form *D* = (1 − *α*/*γ*)/(1 − *α*/*ϕ*), and it can be applied to any admissible measure of diversity for which *α* is defined. *D* was suggested by Jost [32] for Rényi-diversity of order 2 and under the stipulation of equal community sizes. The stipulation of equal community sizes implies *ϕ* = *N* ∙ *α* for Rényi-diversities, so that *D* = (1 − 1/*β*)/(1 − 1/*N*), where *N* is the number of communities and *β* = *γ*/*α* [32, Eq. 10].

It may be tempting to interpret indices such as *D* in terms of partitioning diversity within and between communities, where the extreme conditions appear when “all diversity resides within communities” and when “all diversity resides between communities”. Passing over the fact that “diversity between communities” conflicts with the concept of diversity, the phrasing “all diversity resides between communities” does not address the situation of complete differentiation. Complete differentiation does not exclude the existence of diversity within communities, in which case one could end up with the confusing statement that all diversity resides between but some within communities. Thus “all diversity resides between communities” implies that there is no diversity within communities (all communities are monomorphic), including the possibility that types are shared between communities and that therefore differentiation is not complete.

In the previous section on the duality of differentiation, it was pointed out that this view of partitioning variation is covered by the notion of apportionment of variation to communities rather than of differentiation between them. Indeed, this difference in notion matters especially in connection with the indices *F*_*ST*_ and *G*_*ST*_, which constitute almost the standard for quantifying subpopulation structure in population genetics. They are usually addressed as measures of differentiation among populations but are sometimes also referred to as measures of fixation (genetic monomorphy). The reference to differentiation has been criticized repeatedly, starting with the creator of *F*_*ST*_ himself, Sewall Wright ([33]; see also [34] and, more rigorously, [32]). Yet, as Wright emphasized right from the beginning (and as was recalled to memory by several authors later), *F*_*ST*_ = 1 only in situations of complete monomorphy in all populations and *F*_*ST*_ = 0 only in the absence of differentiation (the same holding for its generalization *G*_*ST*_). Hence, *G*_*ST*_ should more appropriately be addressed as a measure of *partitioning-oriented apportionment*.

The extent to which this confusion of notions and categories of variation may have misled the analysis of models and of experimental results is currently hard to judge. Jost [32] presented some disquieting examples. Even addressing *G*_*ST*_ as an index of fixation is questionable unless a meaningful definition is given of the opposite extreme of “complete fixation”, that is, of complete absence of fixation. Since any degree of absence of fixation is reached by any degree of polymorphism, it is, however, hard to imagine how such a definition could exist. Therefore, to prevent misconceptions triggered by ambivalent terminology, measures such as *G*_*ST*_ should be listed under the notion of partitioning-oriented apportionment, as suggested above. This could also help to simplify decisions about which measure is more appropriate for analyzing “the causes or consequences of population structure” [35].

In any case, the possibly most efficient and comprehensive means of keeping the two approaches to the partitioning of variation apart is to recall the above characterizations, according to which differentiation describes the tendency of individuals of the same type to occur in the same community, and apportionment describes the tendency of individuals of different type to occur in different communities.

The example in Fig. 6 does not represent extreme distributions of types and community memberships, but yet it reveals distinct assessments of the apportionment and differentiation within the category of partitioning variation as indicated by the corresponding descriptors *D* and *G*_*ST*_. It demonstrates that the classical focus on the apportionment approach to partitioning variation may miss important information on its differentiation aspect. Beyond this, it also substantiates the considerable differences that may exist between categories of variation when comparing the TC perspective of compositional differentiation with the CT perspective of partitioning-oriented apportionment on the basis of the $\Delta_{SD}^{TC}$ and *G*_*ST*_ values.

Of course, the dual perspective introduced in connection with compositional differentiation can likewise be applied to all features of the partitioning of variation. Since it again simply follows from switching the variables *T* and *C*, it will not be considered in more detail in this paper.

### Partitioning diversity into components

The term “partitioning” may relate to the decomposition of a set into disjunct subsets or to the decomposition of one variable into several other variables (such as *γ* into *α* and *β*). Between these two aspects, the partitioning of a variable, though less intuitive, is more popular and therefore deserves some explanation. The decomposition of a (initial) variable into additional (component) variables requires the existence of objects for which the states of all variables can be scored. The set of component variables may then be considered to establish a decomposition of the initial variable, if any pair of objects differing in the initial variable also differ in at least one of the component variables. It follows that the states of the initial variable can be considered as a function (in the mathematical sense) of the states of the component variables. The underlying set of objects determines which combination of the states of the component variables are realized and are thus “admissible”. In the present situation, the objects are metacommunities, the initial variable is their *γ*-diversity, and the component variables are the *α*- and *β*-diversity of the metacommunities. A special feature of the (component) variable *α* is that its values obey the inequality *α* ≤ *γ* (which transforms e.g. to *β* ≥ 1 when diversity effective numbers are considered) for all admissible combinations of *α*- and *β*-values.

A fourth variable, the joint diversity *ϕ*, is required when defining partitioning-oriented measures of differentiation that consider variable community sizes. This variable is not an explicit part of the classical concept of partitioning (total type) diversity into components. Its relationship to differentiation indeed suggests that it could be addressed as some kind of *β*-diversity. Yet by the above explanations this requires proof of its eligibility as a component of *γ*-diversity. Given *α*-diversity as the other component it is, however, difficult to see how *ϕ* could explain differences in *γ* that are not explained by *α*. This does of course not rule out the possibility that *ϕ* could qualify as a component of total type diversity in combination with some variable other than *α*. Pertaining studies do however not seem to exist. It thus appears that the role of differentiation in defining components of diversity is currently not well understood. This, in turn, sheds doubts on the role of classical *β*-diversity as a component of total type diversity that accounts for the effects of differentiation (see also [28]).

Problems of dependence or relatedness among components of diversity are discussed by Chao et al. [36]. In their discussions, the terms “dependence” or “relatedness” refer solely to relationships between component variables. These relationships are determined by what is called above “admissible combinations of states of the input variables”, and these are in turn specified by the set of objects under consideration. One can therefore define admissible states for each variable separately by the existence of at least one object that shows the state. The component variables are then defined to be independent or unrelated, if for any choice of admissible states of the individual variables there is an object that realizes this combination of states (so that the combination is again admissible). Given this definition, it is easily verified that in the classical setting, where *β* is specified as a function of *γ* and *α*, the *α*- and *β*-component of diversity are independent, as stipulated by Jost [16]. This statement is not invalidated by the above critique of *β*-diversity as a component that reflects the effects of differentiation.

## Concluding remarks

The present approach is based on the elementary ingredients of variation, i.e., frequencies (or, more general, representations) of types and the differences between them. It is shown that this approach allows descriptors of variation of qualitative traits to be consistently extended to quantitative traits. The basic classification of descriptors of variation within communities into the categories of dispersion and diversity follows from this extension. Both of these categories for within-community variation can be used in the measurement of differentiation among communities, however with emphasis on different aspects relating to the categories of compositional differentiation (differences in trait distribution) and of the partitioning of variation among communities.

Usage of the term “differentiation” in both of the categories compositional differentiation and partitioning of variation is probably unfortunate, since it might obscure the intrinsic difference between the two categories. This difference is emphasized in the introductory sentences of the “Partitioning of variation” section, where the prefix “partitioning-oriented” is added to differentiation as a reminder that this kind of “differentiation” is to be understood as relating to a component that is only a part of the total variation. Compositional differentiation, in contrast, is defined without any reference to the total variation.

The partitioning of diversity into components is almost always considered as the decomposition of the total diversity (*γ*) in a metacommunity into the contribution of the diversities within the communities (*α*) and a remainder that is thought of as the contribution of the differences between the communities. The latter is commonly addressed as *β*-diversity in ecology. Yet, as a component of total diversity, *β*-diversity (which is not even a proper measure of diversity) remains ambivalent as to what it represents. Common measures (all of which are functions of *γ* and *α*-diversity) do not unequivocally accord with the concept of differentiation but instead reflect tendencies towards monomorphy. As a “component” of total diversity, the idea of *β*-diversity definitely needs conceptual reconsideration.

Among the innumerable applications of the present classification, some have already been demonstrated above. The following two deserve special mention because of their broader implications.

Effective numbers are characteristic of diversity descriptors, but they may also serve to quantify the number of types that support an observed dispersion, provided the latter is based on a measure of dissimilarity. Descriptors of dispersion that are not based on dissimilarity measures cannot be traced back to (effective) numbers of types. Even though special measures of dispersion do obey the evenness criterion and thus are also measures of diversity (such as the Simpson index), one should be aware of the essential difference when interpreting them as descriptors of diversity or of dispersion.

Because of its broad interest and varying terminological usage (see e.g. [37]), a brief final remark seems appropriate on how phylogenetic diversity fits into the present classification of biological variation. The reconstruction of phylogenies relies on measures of difference or similarity that indicate relatedness by common descent; as such they serve the reconstruction of monophyletic groups (clades) that are hierarchically organized (i.e., form an encaptic set structure). Such a reconstruction is compatible with the difference measure (making it a phyletic distance), if the differences between members of a clade are always smaller than their differences from organisms that do not belong to this clade. For each threshold value of difference, this condition allows the formation of a unique partition with clades as classes, in which members of the same class differ by not more than the threshold value while members of different classes differ by more. These partitions are commonly used for the identification of taxonomic categories or ranks. Descriptors of phylogenetic diversity are then consistently defined for each such partition, as explained above in connection with clustering methods (see also [38]). The distribution of phylogenetic diversity across ranks can then be illustrated and analyzed with the help of diversity portraits.

## Appendix A: Glossary

Trait: A set of mutually exclusive attributes of the members of a collection of objects. The individual attributes define the states of the trait. For more detailed explanation of the trait concept, see [39]. Traits may be of **qualitative** nature (i.e., the states of two objects are either the same or different, such as specification of the species in a systematic category, the alleles at a gene locus, community membership in a metacommunity, etc.) or of **quantitative** nature (i.e., states can differ by varying amounts, e.g. metric amounts such as height, length, diameter, etc.). For the trees in a central European forest community, for example, the trait “species” has states *Fagus sylvatica*, *Quercus robur*, *Q. petraea*, *Picea abies*, among many others. The qualitative trait “species” can be made quantitative by assigning each pair of species a phylogenetic distance, which usually stipulates that species from the same genus (e.g. Quercus) are more similar to each other than to species from different genera. The qualitative trait “genotype at a specified gene locus” with states “*A*_1_ *A*_1_”, “*A*_1_ *A*_2_”, etc. can be made quantitative by specifying the difference between states as the proportion 0, $\frac{1}{2}$ or 1 of alleles that the respective genotypes do not share at this locus.
Community: Any collection or ensemble of individuals that are connected and delineated by specified ecological or reproductive factors (including functional species communities, populations as reproductive communities, etc.)
Metacommunity: Totality of all individuals in any of a specified set of communities
Type frequency: Relative quantitative representation of the presence of a type (or trait state) in a community, often as the relative frequency of the individuals that carry the type
Difference between types: Assessment of **qualitative difference** (same or different, e.g. species of a systematic category, alleles at a gene locus, community membership in a metacommunity) or **quantitative difference** (usually metric measurements, e.g. height, proportion of non-shared alleles) between the types of two individuals
Norm of reaction: A norm of reaction is defined for any one genetic type and a trait in the expression of which the genetic type is involved. The differential effects of environmental conditions on the expression of the trait can then be described by a mapping of these conditions onto the trait expressions. This mapping is usually referred to as the reaction norm of a genetic type (with respect to a specified trait and set of environmental conditions).
Genotype-environment association: When genotypes are distributed at random over environmental conditions, there is no association between them. Otherwise there is.
Genotype-environment interaction: Generally describes the failure to separate genetic from environmental effects on a specified trait. This situation typically arises when norms of reaction intersect, i.e. when two distinct genetic types express the same trait state in the same environment.
Aspects of biological variation within communities:
  Dispersion: Assessment of the spread of the differences between types in a community (e.g. maximum difference, variance)
  Diversity: Assessment of the number of types or clusters of types in a community, usually with regard to their representation in terms of frequencies or relative sizes, respectively. The **intrinsic concept of diversity** addresses numbers of types irrespective of their frequencies (commonly referred to as “richness”). Diversity measures fulfill the evenness criterion, in that they never decrease whenever the difference in representation between two types decreases while the sum of their representations remains the same.
Aspects of the distribution of biological variation over communities:
  Compositional differentiation: Assessment of differences between ***c***ommunities for the distribution of the ***t***rait states of a specified trait (**CT perspective**) or assessment of differences between ***t***rait states for their distributions over ***c***ommunities (TC perspective)
  Partitioning of variation: Assessment of the partition of the total (metacommunity) variation into variation within and between communities. There are two approaches: **differentiation** describes the extent to which individuals with the same trait state or type occur in the same community, and **apportionment** describes the extent to which individuals with different types occur in different communities.

## Appendix B: Proof of the evenness criterion

**Lemma:** Let *g* be a real-valued function defined on a closed interval, and let *c* be a number from the interior of the interval. If *g* is (not necessarily strictly) concave then *f*(*x*): = *g*(*x*) + *g*(*c* − *x*) increases (not necessarily strictly) as *x* approaches *c*/2 from above or below.

**Proof:** Consider *f*(*x*′) − *f*(*x*) = *g*(*x*′) − *g*(*x*) + *g*(*c* − *x*′) − *g*(*c* − *x*) with all arguments in the interval of definition. Concavity implies that [*g*(*x*′) − *g*(*x*)]/[*x*′ − *x*] is monotonically non-increasing in *x*′ for fixed *x* and *x*′ ≠ *x* (non-increasing slope). For *x < x*′ ≤ *c*/2, and therefore *x*′ ≤ *c* − *x*′, one thus obtains [*g*(*x*′)−*g*(*x*)]/[*x*′−*x*] ≥ [*g*(*c*−*x*′)−*g*(*x*)]/[*c*−*x*′−*x*], or [*g*(*x*′)−*g*(*x*)] ≥ [*g*(*c*−*x*′)−*g*(*x*)]·[*x*′−*x*]/[*c*−*x*′−*x*]. By the same means, since *x* ≤ *c* − *x*, [*g*(*c* − *x*′) − *g*(*x*)]/[*c* − *x*′ − *x*] ≥ [*g*(*c* − *x*′) − *g*(*c* − *x*)]/[*x* − *x*′] or [*g*(*c* − *x*′) − *g*(*c* − *x*)] ≥ [*g*(*c* − *x*′) − *g*(*x*)] · [*x* − *x*′]/[*c* − *x*′ − *x*]. Hence,

$$f\text{(}x^{\prime }\text{)}-f\text{(}x\text{)}\geq \text{[}g\text{(}c-x^{\prime }\text{)}-g\text{(}x\text{)]}\cdot \frac{x^{\prime }-x}{c-x^{\prime }-x}+\text{[}g\text{(}c-x^{\prime }\text{)}-g\text{(}x\text{)]}\cdot \frac{x-x^{\prime }}{c-x^{\prime }-x}=0$$

Since *f*(*x*) = *f*(*c* − *x*) it follows analogously that for *c*/2 ≤ *x*′ < *x*, *f*(*x*′) ≥ *f*(*x*). QED

**Proposition:** If *r* is a real-valued function defined on the unit interval, and if *p* · *r*(*p*) is concave, then Σ_*i*_ *p*_*i*_ · *r*(*p*_*i*_) fulfills the evenness criterion.

**Proof:** Let *p*_*i*_ + *p*_*j*_ = *c*, and set *p* = *p*_*i*_, *p*_*j*_ = *c* − *p* and *g*(*p*) = *p* · *r*(*p*). Then the above Lemma applies, and Σ_*i*_ *p*_*i*_ · *r*(*p*_*i*_) increases as |*p*_*i*_ − *p*_*j*_| decreases while *p*_*i*_ + *p*_*j*_ = *c*. QED—Also compare the pertaining results of Patil & Taillie (1982, p.551).

**Note:** *p* · *r*(*p*) is concave, for example, if *r* is a decreasing and concave function of *p*.
